# Effect of the ratios of estradiol increase on the outcome of in vitro fertilization-embryo transfer with antagonist regimens: a single center retrospective cohort study

**DOI:** 10.1186/s12884-023-05438-3

**Published:** 2023-03-02

**Authors:** Chun-Xiao Wei, Liang Zhang, Cong-Hui Pang, Ying-Hua Qi, Jian-Wei Zhang

**Affiliations:** 1 Shandong University of Traditional Chinese Medicine Affiliated Hospital. Jinan, Shandong, China; 2 Shandong University of Traditional Chinese Medicine. Jinan, Shandong, China

**Keywords:** Estradiol level, Increase ratio, IVF, Antagonist regimen, Cumulative live birth rates

## Abstract

**Background:**

The outcome of in vitro fertilization-embryo transfer (IVF) is often determined according to follicle and estradiol levels following gonadotropin stimulation. In previous studies, although most of them analyzed the estrogen level from ovaries or the average estrogen level of a single follicle, there was no study on the ratio of estrogen increase, which was also correlated with pregnancy outcomes in the clinic. This study aimed to make timely adjustments to follow-up medication to improve clinical outcomes based on the potential value of estradiol growth rate.

**Methods:**

We comprehensively analyzed estrogen growth during the entire ovarian stimulation period. Serum estradiol levels were measured on the day of gonadotropin treatment (Gn1), five days later (Gn5), eight days later (Gn8), and on the trigger day (HCG). This ratio was used to determine the increase in estradiol levels. According to the ratio of estradiol increase, the patients were divided into four groups: A1 (Gn5/Gn1 ≤ 6.44), A2 (6.44 < Gn5/Gn1 ≤ 10.62), A3 (10.62 < Gn5/Gn1 ≤ 21.33), and A4 (Gn5/Gn1 > 21.33); B1 (Gn8/Gn5 ≤ 2.39), B2 (2.39 < Gn8/Gn5 ≤ 3.03), B3 (3.03 < Gn8/Gn5 ≤ 3.84), and B4 (Gn8/Gn5 > 3.84). We analyzed and compared the relationship between data in each group and pregnancy outcomes.

**Results:**

In the statistical analysis, the estradiol levels of Gn5 (*P* = 0.029, *P* = 0.042), Gn8 (*P* < 0.001, *P* = 0.001), and HCG (*P* < 0.001, *P* = 0.002), as well as Gn5/Gn1 (*P* = 0.004, *P* = 0.006), Gn8/Gn5 (*P* = 0.001, *P* = 0.002), and HCG/Gn1 (*P* < 0.001, *P* < 0.001) both had clinical guiding significance, and lower one significantly reduced the pregnancy rate. The outcomes were positively linked to groups A (*P *= 0.036, *P* = 0.043) and B (*P* = 0.014, *P* = 0.013), respectively. The logistical regression analysis revealed that group A1 (OR = 0.376 [0.182–0.779]; *P* = 0.008*, OR = 0.401 [0.188–0.857]; *P* = 0.018*) and B1 (OR = 0.363 [0.179–0.735]; *P* = 0.005*, OR = 0.389 [0.187–0.808]; *P* = 0.011*) had opposite influence on outcomes.

**Conclusion:**

Maintaining a serum estradiol increase ratio of at least 6.44 on Gn5/Gn1 and 2.39 on Gn8/Gn5 may result in a higher pregnancy rate, especially in young people.

## Introduction

During in vitro fertilization (IVF) cycles, controlled ovarian hyperstimulation (COH) treatment using exogenous gonadotropin (Gn) to stimulate follicle development is a critical step in ensuring the acquisition of mature eggs and a satisfactory pregnancy rate. In clinical practice, assisted reproductive technology (ART) outcomes are often monitored according to the size and number of follicles and serum estradiol (E_2_) levels after Gn stimulation. However, no accurate indicator to predict pregnancy outcomes has been identified to date, and it has not been determined how to choose subsequent drugs and doses based on the ovarian response after stimulation.

Additionally, the need for combined monitoring (using transvaginal ultrasound and serum estradiol) during ovarian stimulation is controversial. Some people argued that vaginal ultrasound alone should be considered to simplify treatment, as combined monitoring is costly, time-consuming, and inconvenient [1]. Neither E_2_ on the day of human chorionic gonadotropin (hCG) administration nor other stages were linked to pregnancy rates in women undergoing ART cycles [2–5]. Additionally, E2 level was found to be a poor predictor of treatment success [6]. However, the evidence has a low overall quality [7]. Additionally, other researchers have suggested that E_2_ levels can be used to predict pregnancy outcomes in combination with FSH, age, inhibin B, and other factors [8–11]. Some researchers have suggested that a poor ovarian response can be characterized by peak E_2_ levels [12]. Phelps et al. and Kahyaoglu et al. [13, 14] explored the relationship between E_2_ levels on the fourth day of Gn and IVF outcomes and believed that estradiol levels on the fourth day of COH cycle could predict the response of early follicles to ovarian stimulation. When the serum estradiol level on the fourth day of Gn was low, the current treatment cycle was abandoned. Other groups concluded that a low E_2_ concentration after five days of Gn stimulation predicted a high cycle cancellation and lower pregnancy outcome, even with similar numbers of oocytes and fertilization rates [15]. Lower E_2_ levels on the sixth day of Gn have also been associated with a lower pregnancy rate and the likelihood of live births [8, 16, 17]. It has been reported that an appropriate range for E_2_ exists, and a higher range is not beneficial [18, 19]. Older women (> 35 years) appeared to be more vulnerable to the harmful effects of high E_2_ levels than younger women (≤ 35 years). Valbuena et al. [20, 21] found that high E_2_ concentration affects embryonic adhesion. Additionally, high E_2_ concentration affects endometrial receptivity [22–25]. However, Blazar et al. [26–28] discovered that higher E_2_ levels on hCG days predicted a greater number of oocytes, and any adverse impact on endometrial can be conquered in IVF-ET. Only the estradiol level on the hCG day was a significant predictive variable for clinical pregnancy [29, 30]. Additionally, according to percentile curves, Papageorgiou et al. [31] did not identify any deleterious effects of high E_2_ levels. Super physiological estradiol levels also do not affect oocyte and embryo quality [32].

Everyone had different opinions on determining E_2_ in COH cycle, but it was undeniable that determining E_2_ during follicular phase has become a part of routine clinical practice over the last decade. In previous studies, most of them only analyzed the estrogen level, and there was no study on the ratio of estrogen increase, although different people had different antral follicle counts and different average estrogen levels of a single follicle. Estrogen levels represent the static condition of the ovarian response and do not reflect the dynamic state of exogenous control of ovarian stimulation on follicular growth. Thus, this study aimed to determine whether serum E_2_ levels and E_2_ increase ratios during Gn ovarian stimulation correlated with IVF and pregnancy outcomes in 335 patients on antagonist regimens. Additionally, this study aimed to determine the link between E_2_ levels and ratios in the whole Gn stimulation period and the outcomes, and the number of embryos was investigated to distinguish E_2_ effects on the endometrium and embryos. If this hypothesis is substantiated, it may be time to seek a new method to evaluate the ovarian response during Gn stimulation, adjust the dose, and ensure treatment outcome of COH.

## Materials and methods

### Study subjects and protocol

From April 2017 to July 2020, our center conducted a retrospective analysis of infertile patients who underwent IVF cycles. The women were monitored until live birth or until the embryos in the current cycle had been completed. The study protocol was approved by the institutional human ethics committee.

Inclusion criteria were as follows: 1) age ≤ 38 years, 2) basal follicle-stimulating hormone (bFSH) ≤ 10 IU/L, basal antral follicle count (bAFC) > 3, and 3) body mass index (BMI) < 30 kg/m^2^.

Exclusion criteria were as follows: 1) patients with chromosomal abnormalities, reproductive malformation, and a history of recurrent spontaneous abortion; 2) patients undergoing coasting to prevent ovarian hyper stimulation syndrome; 3) insufficient information; 4) uterine pathologies that might compromise pregnancy potential; and 5) cycles canceled due to failure of embryo thawing and survival.

### Ovarian stimulation protocol

Patients received IVF treatment according to the Fixed GnRH antagonist protocol [33]. On the second day of the menstrual cycle, recombinant human follicle-stimulating hormone 150–300 U (Gonal-F; Merck, Lyon, France; Puregon, MSD, Boulogne, France) was injected as Gn. Additionally, Gn doses were determined based on patient age, body mass index (BMI), bFSH, and bAFC. GnRH-ant (Cetrotide, Merck, Lyon, France) was administered from day 5 onward. Oocytes were then collected by follicular aspiration under ultrasound 34–36 h after triggering with GnRH-a (Triptoreline, Decapeptyl, Ipsen, France) or recombinant hCG (rhCG, Ovitrelle, Merck, Lyon, France). Eighteen hours after fertilization, embryo development was monitored daily and graded based on the number and size of blastomeres, fragmentation rate, multinucleation, and early densification. Notably, on the third day following oocyte retrieval, an embryo with seven–ten blastomeres was defined as high quality [34].

On day 3, one or two embryos in the best shape were selected and transferred using a soft Wallace catheter, or whole embryo freezing for vitrification was chosen to avoid ovarian hyperstimulation syndrome (OHSS), embryo–endometrium asynchrony, and other reasons.

For luteal support in advance of fresh embryo transfer (ET), we used an injection of progesterone (20 mg/branch, Zhejiang Xianju Pharmaceutical Co., Ltd.), 40 mg daily, and oral dydrogesterone tablets (10 mg/tablet, Abbott Healthcare Products B.V.), 30 mg per day, or progesterone vaginal sustained-release gel (90 mg/dose, Crinone VR 8%, Merck, Sherano, Switzerland). In addition, two bags of Chinese medicine, the Gushen Antai pills, were used daily. For the frozen embryo transfer (FET), the endometrial preparation protocols used before the FET were natural cycles, hormone replacement cycles, and stimulated cycles. Embryo transfer was performed after three days of progesterone supplementation. For natural cycles and hormone replacement cycles, progesterone supplementation was added, similar to the cycle of fresh embryo transfer. Stimulated cycle protocols for endometrial preparation were not supplemented with progesterone.

All patients completed an IVF cycle and then performed ET or FET until live birth or until the embryos in the current cycle were used up.

### Calculation of serum E2

Venous blood samples were collected on the day of Gn (Gn1), on five days of Gn (Gn5), on eight days of Gn (Gn8), and the day of trigger (HCG). In addition, Gn5/Gn1, Gn8/Gn5, HCG/Gn1, HCG/Gn5, and HCG/Gn8 represented the ratios of serum E_2_ increase. The ratios were calculated as follows: Gn5 divided by Gn1, Gn8 divided by Gn5, HCG divided by Gn1, HCG divided by Gn5, and HCG divided by Gn8.

### Pregnancy outcomes

Cumulative live birth rate (CLBR) was the primary outcome. Secondary outcomes included cumulative clinical pregnancy rate (CCPR), number of oocytes, number of fertilization, number of blastomeres, number of embryos, and number of high-quality embryos.

CLBR was defined as the first live birth after using fresh or frozen embryos derived from a single ovarian stimulation cycle.

Clinical pregnancy was defined as an intrauterine gestational sac with a fetal heartbeat detected on transvaginal ultrasonography after six weeks of gestation. CCPR was calculated as the number of first clinical pregnancies generated from a single IVF cycle, including all fresh and frozen embryo transfers generated from the IVF cycle.

### Statistical analysis

Statistical analysis was conducted using SPSS version 26.0 (SPSS Inc., Chicago, IL, USA). The Shapiro–Wilk test was used to assess data normality. Due to skewed distributions, quantitative variables were expressed as medians (interquartile range, range between the 25th and 75th percentiles), and Mann–Whitney U and Kruskal–Wallis tests were performed. Qualitative variables were expressed as frequencies and analyzed using chi-square test. *P* ≤ 0.05 was considered statistically significant.

Groups A and B were defined according to the 25th, 50th, and 75th percentiles of each ratio of E_2_ increase. According to the ratio of estradiol increase, the patients were divided into four groups: A1 (Gn5/Gn1 ≤ 6.44), A2 (6.44 < Gn5/Gn1 ≤ 10.62), A3 (10.62 < Gn5/Gn1 ≤ 21.33), and A4 (Gn5/Gn1 > 21.33); B1 (Gn8/Gn5 ≤ 2.39), B2 (2.39 < Gn8/Gn5 ≤ 3.03), B3 (3.03 < Gn8/Gn5 ≤ 3.84), and B4 (Gn8/Gn5 > 3.84).

Spearman’s correlations were used to determine the correlation between the quantitative parameters and the increase in E_2_ levels. The propensity scores were calculated using binary logistic regression analysis based on the following patient characteristics: female age, infertility duration, body mass index (BMI), infertility factors, Gn usage time, and Gn dosage. We calculated crude odds ratios (OR) and adjusted OR with 95% confidence intervals (CI).

## Results

### Study population

From April 2017 to July 2020, we retrospectively analyzed 335 patients who received in vitro fertilization (IVF) with antagonist regimens at the Affiliated Hospital of Shandong University of Traditional Chinese Medicine. Table 1 summarizes the characteristics of the study population for positive and negative CCPR and CLBR results. CCPR positives included 160 patients, and negative s included 175 patients. A total of 124 women had viable CLBR, whereas 211 did not.

**Table 1:** Baseline characteristics for CCPR and CLBR

Parameter	CCPR positive	CCPR negative	*P*-Value	CLBR positive	CLBR negative	*P*-Value
Number of cycles	160	175		124	211	
Age, years	31 (29, 33)	33 (30, 36)	0.001*	31 (29, 33)	32 (30, 36)	0.006*
BMI, kg/m^2^	23.3 (20.7, 26.2)	23.3 (20.7,26.8)	0.880	22.5 (20.5, 26.3)	23.4(20.8,26.5)	0.347
Duration of infertility, years	3(2, 5)	3(2, 5)	0.651	3(2, 4.5)	3(2, 5)	0.580
Gn days	10(9, 11)	10(9, 11)	0.622	10(9, 11)	9(9, 11)	0.773
Gn dosage, IU	2025(1594, 2437)	2250 (1800,2968)	0.001*	2022(1575, 2437)	2212(1725,2875)	0.005*
Number of embryo transfers	2 (1, 3)	2 (1, 2)	0.146	2 (1, 3)	2 (1, 2)	0.158
Baseline FSH, IU/L	7.29(6.30, 8.67)	7.66 (6.26, 10)	0.102	7.49(6.20, 8.66)	7.40 (6.32, 9.89)	0.522
Baseline LH, IU/L	4.88 (3.78, 7.70)	4.58(3.44, 5.71)	0.246	5.00 (3.53, 7.05)	4.73(3.50, 6.54)	0.316
Baseline E_2_, pg/mL	40.00(30.00, 55.00)	45.00(32.00, 59.00)	0.134	40.00(30.00, 54.50)	44.00(31.50, 59.00)	0.122
AFC	33.00(23.00,46.00)	22.00(15.00,32.00)	< 0.001*	30.00(22.00,43.00)	24.00(17.00,46.00)	0.001*
endometrial thickness, mm	11.05 (9.80, 12.58)	11.00 (9.82, 12.50)	0.929	11.20 (9.68, 12.93)	11.00 (9.98, 12.50)	0.570
Number of oocytes	12(9,20)	9(5, 14)	< 0.001*	13(9,19.5)	10(6, 14)	< 0.001*
Number of fertilization	7 (4, 11)	5 (2, 9)	< 0.001*	7 (4, 11)	5 (2, 9)	< 0.001*
Number of blastomere	6 (4, 10)	4 (2, 8)	< 0.001*	6 (4, 10)	5 (2, 8)	< 0.001*
Number of embryos	4 (3, 6)	2(1, 4)	< 0.001*	4(3, 6)	2(2, 4)	< 0.001*
Number of high-quality embryos	1(0, 2)	0 (0, 1)	< 0.001*	1(0, 3)	0 (0, 1)	< 0.001*

Values are given as median (range)

Abbreviations: *BMI * Body mass index, *Gn days * Gonadotropin days, *Gn dosage* Gonadotropin dosage, *CCPR * Cumulative clinical pregnancy rate, *CLBR * Cumulative live birth rate, *AFC* Antral follicle count

### Baseline characteristics

Positive and negative results were similar for BMI, infertility duration, Gn days, number of embryos transferred, endometrial thickness on transplantation day, and baseline hormone levels.

However, patients with CCPR and CLBR positive results were younger than those with negative results (*P* = 0.001 and *P* = 0.006, respectively) and had a lower Gn dosage (*P* = 0.001 and *P* = 0.005)). The patients with CCPR and CLBR positives had more antral follicle counts (*P* < 0.001 and *P* = 0.001). The patients with CCPR and CLBR positives achieved better IVF-ET outcomes (both *P* < 0.001), such as the number of oocytes, fertilization, blastomere, and embryos.

### Serum estradiol levels and ratios of CCPR and CLBR

Table 2 compares the outcomes based on E_2_ levels and ratios. Gn1 did not impact IVF outcomes (*P* = 0.134; *P* = 0.122). However, elevated E_2_ levels following gonadotropin stimulation correlated with higher CCPR and CLBR (both *P* < 0.05), particularly in the late follicular phase, E_2_ of Gn8 (*P* < 0.001; *P* = 0.001), and HCG (*P* < 0.001; *P* = 0.002). However, in early follicular growth, the estrogen increase ratios were more statistically significant with the outcomes. Following gonadotropin stimulation, the higher the serum estradiol ratios of Gn5/Gn1 (group A) and Gn8/Gn5 (group B), the higher the CCPR (*P* = 0.004; *P* = 0.001) and CLBR (*P* = 0.006; *P* = 0.002, respectively). In contrast, estrogen ratios of HCG/Gn5 and HCG/Gn8 in late follicle growth were similar across groups without reaching statistical significance.

**Table 2:** The level and the ratio of serum E_2_ for CCPR and CLBR

	CCPR positive	CCPR negative	*P*-Value	CLBR positive	CLBR negative	*P*-Value
E_2_(Gn1), pg/mL	40(30, 55)	45(32, 59)	0.134	40(30, 54.5)	44(31.5, 59)	0.122
E_2_(Gn5), pg/mL	572.00(308, 1067.5)	447(246.5, 782)	0.029*	594 (321,1025)	448(246.5, 6796.5)	0.042*
E_2_(Gn8), pg/mL	1889(1089,3452)	1315(734.5, 2038)	< 0.001*	1889(1093,3280)	1432(785.5, 2281)	0.001*
E_2_(HCG), pg/mL	4139(2243.5, 5010)	2720(1552, 4775)	< 0.001*	4166(2243,4996)	2796(1646, 4800)	0.002*
E_2_ ratio of Gn5/Gn1	12.2(7.38, 28.97)	9.88(5.41, 17.52)	0.004*	12.93(7.44, 28.92)	9.96(5.59, 18.9)	0.006*
E_2_ ratio of Gn8/Gn5	3.14(2.6, 3.99)	2.83(2.14, 3.73)	0.001*	3.24(2.63, 4.05)	2.87(2.27, 3.75)	0.002*
E_2_ ratio of HCG/Gn1	86.38(47.05, 129.7)	58.76(31.93, 100.6)	< 0.001*	87.22(47.48, 131.48)	59.73(34.47, 102.12)	< 0.001*
E_2_ ratio of HCG/Gn5	5.43(3.3, 8.29)	4.93(3.17, 8.63)	0.514	5.64(3.32,8.39)	4.99(3.22, 8.44)	0.562
E_2_ ratio of HCG/Gn8	1.57(1.15, 2.33)	1.70(1.14, 2.64)	0.113	1.62(1.18, 2.39)	1.66(1.13, 2.54)	0.245

Values are given as median (range)

Abbreviations: *E*_*2*_ Estradiol, *Gn1* Serum estradiol on the day of gonadotrophin, *Gn5* Serum estradiol on the five day after gonadotropin stimulation, *Gn8* Serum estradiol on the eight day after gonadotropin stimulation *HCG * Serum estradiol on the trigger day of human chorionic gonadotropin injection, *Gn5/Gn1 * The serum estradiol levels of Gn5 divided by Gn1, *Gn8/Gn5 * The serum estradiol levels of Gn8 divided by Gn5, *HCG/Gn1 * The serum estradiol levels of HCG divided by Gn1, *HCG/Gn5 * The serum estradiol levels of HCG divided by Gn5, *HCG/Gn8 * The serum estradiol levels of HCG divided by Gn8

### Parameters of different serum estradiol ratios

According to Table 3, data were classified into four groups based on the quartile of serum E_2_ ratio. As displayed in Table 3, although group A exhibited no significant differences in terms of female age, BMI, and infertility time, group B indicated significant differences (both *P* < 0.05). For group A, the higher the estrogen growth rate, the shorter the Gn time required and the lower the Gn dose used (both *P* < 0.001), whereas for group B, the opposite was true (both *P* < 0.005). However, when estrogen growth rate increased, groups A and B produced superior IVF-ET outcomes in terms of the number of embryos harvested, blastomeres, embryos, CCPR, CLBR, etc. (both *P* < 0.05). The ratio in group A increased as the ratio in group B decreased (*P* < 0.001). When the ratio in group B was larger, the corresponding ratio in group A was smaller. When the values of the two groups were higher, E_2_ levels on the hCG day were also higher (both *P* < 0.001).

**Table 3:** Parameters and outcome of IVF-ET as a function of serum E_2_ ratios

	A1	A2	A3	A4	P-Value	B1	B2	B3	B4	*P*-Value
Number of cycles	84	84	84	83		84	84	84	83	
Age, years	32(29,34)	31.5(29,34)	31(30,35)	33(30,35)	0.61	33(30,36)	32(30,35)	32(29,34)	31(29,33)	0.044*
BMI, kg/m^2^	22.60(20.70,26.20)	23.05(20.40,26.40)	23.40(20.40,27.00)	24.00(21.70,27.30)	0.41	25.40(22.00,29.05)	22.80(21.35,25.85)	22.20(20.20,26.20)	23.25(20.40,26.00)	0.002*
Duration of infertility, years	3(2,4)	3(1,5)	3(2,5)	3(1,5)	0.59	4(2,6)	3(2,4.5)	3(2,4)	3(1.5,4)	0.01*
Gn days	11(10,12)	10(9,11)	9(8,10)	9(8,10)	< 0.001*	9(8,10)	9(9,11)	10(9,11)	10.5(10,11)	< 0.001*
Gn dosage, IU	2700(2250,3300)	2250(1725,2625)	2025(1725,2400)	1770(1500,2100)	< 0.001*	1900(1500,2400)	2175(1812,2700)	2175(1650,2550)	2370(1975,2910)	0.004*
endometrial thickness, mm	10.50(9.30, 12.30)	11.05(9.55, 12.90)	11.00(9.65, 12.70)	11.50(10.50, 12.50)	0.102	11.00(9.80, 12.40)	10.90(10.00, 12.30)	11.20(10.20, 12.50)	11.40 (9.40, 12.90)	0.879
Number of eggs	8(4,12)	10(6,15)	10(8,16)	16(12,23)	< 0.001*	9(5,14.5)	11(6,16)	10(8,14)	14.5(10,20.5)	< 0.001*
Number of fertilizatin	4(2,7)	6(3,9)	6.5(4,11)	8(6,13)	< 0.001*	5(2,8)	6(3,9)	6(4,9)	8(6,13)	< 0.001*
Number of blastomere	3(2,6)	5(3,8)	6(4,10)	7(5,12)	< 0.001*	4(2,7)	5(3,8.5)	5(3,8)	7(4,11)	0.002*
Number of embryos	2(1,4)	4(2,6)	4(2,6)	5(3,8)	< 0.001*	3(2,5)	3(2,5.5)	4(2,6)	4(2,6.5)	0.005*
Number of high-quality embryos	0(0,1)	1(0,2)	1(0,2)	1.5(0,3)	< 0.001*	1(0,2)	1(0,1)	1(0,3)	1.5(0,3)	0.03*
CCPR	32/84(38.1%)^a^	40/84(47.6%)	38/84(45.2%)	50/83(60.2%)	0.036*^a^	30/84(35.7%)^c^	36/84(42.9%)	47/84(56.0%)	47/83(56.6%)	0.014*^c^
CLBR	21/84(25.0%)^b^	33/84(39.3%)	32/84(38.1%)	38/83(45.8%)	0.043*^b^	22/84(26.2%)^d^	26/84(31.0%)	39/84(46.4%)	37/83(44.6%)	0.013*^d^
E_2_ ratio of Gn5/Gn1	–	–	–	–		15.28(7.36,32.33)	12.85(7.85,25.94)	9.40(5.12,17.43)	9.19(5.67,14.91)	< 0.001*
E_2_ ratio of Gn8/Gn5	3.28(2.70,4.04)	3.15(2.32,4.18)	3.12(2.55,4.08)	2.70(2.08,3.15)	< 0.001*	–	–	–	–	
E_2_(HCG), pg/mL	2166(1148,3616)	2592(1589,4800)	3176(2197,4800)	4870(3904,5010)	< 0.001*	2251(1277,4800)	3212(1895,4870)	2989(1920,4800)	4410(2758,5010)	< 0.001*

Values are given as number (percentage) or median (range)

Group A1 (ratio of Gn5 to Gn1 ≤ 6.44), group A2 (6.44 < ratio of Gn5 to Gn1 ≤ 10.62), group A3 (10.62 < ratio of Gn5 to Gn1 ≤ 21.33), group A4 (ratio of Gn5 to Gn1 > 21.33); group B1 (ratio of Gn8 to Gn5 ≤ 2.39), group B2 (2.39 < ratio of Gn8 to Gn5 ≤ 3.03), group B3 (3.03 < ratio of Gn8 to Gn5 ≤ 3.84), group B4 (ratio of Gn8 to Gn5 > 3.84)

^a ^In the pairwise comparison of group A in CCPR, the comparison between group A1 and group A4 was statistically significant (*P* = 0.004)

^b ^In the pairwise comparison of group A in CLBR, the comparison between group A1 and group A2 was statistically significant (*P* = 0.047), and group A1 and group A4 was statistically significant (*P* = 0.005)

^c ^In the pairwise comparison of group B in CCPR, the comparison between group B1 and group B3 was statistically significant (*P* = 0.008), and group B1 and group B4 was statistically significant (*P* = 0.007)

^d ^In the pairwise comparison of group B in CLBR, the comparison between group B1 and group B3 was statistically significant (*P* = 0.006), and group B1 and group B4 was statistically significant (*P* = 0.013)

### Serum estradiol ratio of different ages

As illustrated in Table 4, there were 252 patients in the < group with age below 35 years old and 83 in the other group. The number of eggs obtained and high-quality embryos were statistically significant with the different E_2_ ratios of Gn5/Gn1, Gn8/Gn5 and HCG/Gn1 in both groups with age < 35 and age ≥ 35 (both *P* < 0.001). For people with age < 35, the higher ratios of Gn8/Gn5 and HCG/Gn1 could achieve the higher CCPR and CLBR (both *P* < 0.05). In contrast, the difference was not statistically significant with the E_2_ ratio of Gn5/Gn1. For people with age ≥ 35, the CCPR and CLBR were higher with the E_2_ ratio of HCG/Gn1. However, the differences were insignificant with Gn5/Gn1 and Gn8/Gn5.

**Table 4:** Relationship between the ratio of serum E_2_ and the outcome according to age

	*P*-Value (Age, years < 35)				*P*-Value (Age, years ≥ 35)			
	Number of eggs	Number of high-quality embryos	CCPR	CLBR	Number of eggs	Number of high-quality embryos	CCPR	CLBR
E_2_ ratio of Gn5/Gn1	< 0.001*	< 0.001*	0.413	0.404	< 0.001*	< 0.001*	0.467	0.149
E_2_ ratio of Gn8/Gn5	0.002*	0.004*	0.004*	< 0.001*	< 0.001*	< 0.001*	0.653	0.207
E_2_ ratio of HCG/Gn1	< 0.001*	< 0.001*	< 0.001*	< 0.001*	< 0.001*	< 0.001*	0.008*	< 0.001*

Values are given as median (range)

### Serum estradiol ratio of different infertility factors

Infertility factors included tubal factors (231, 68.9%), ovulation disorders (64, 19.1%), male factors (27, 8.1%), pelvic endometriosis (8, 2.4%), and other factors (5, 1.5%). Table 5 indicates that groups A and B did not correlate with varying infertility factors.

**Table 5:** Relationship between serum E_2_ increase and infertility factors

	A				χ2	*P*	B				χ2	*P*
	A1	A2	A3	A4			B1	B2	B3	B4		
Tubal factors	60	66	54	51	16.788	0.158	56	59	64	52	10.625	0.561
Ovulation disorders	13	13	18	20			16	17	9	22		
Male factors	9	5	7	6			7	5	7	8		
Pelvic endometriosis	1	0	3	1			2	1	2	0		
Other factors	1	0	2	5			3	2	2	1		

χ2-test

### Estradiol levels and ratios of different ovarian responses

Estradiol levels and ratios of different ovarian responses were displayed in Table 6. We divided the population into three parts according to the number of oocytes retrieved, high ovarian responders (> 18 oocytes retrieved), low ovarian responders (< 6 oocytes retrieved), and normal ovarian responders groups. After analysis respectively, just the E_2_ ratio of Gn8/Gn5 had statistically significant. For the low ovarian response and normal ovarian response, the CLBR positive one had higher E_2_ ratio (*P* < 0.001; *P* = 0.017, respectively). In the high response group, the E_2_ ratio with CLBR positive was lower than that with CLBR negative (*P* = 0.004). However due to the small sample size post-treatment, this result requires further verification.

**Table 6:** The level and the ratios of serum E_2_ for different ovarian responses

	Low ovarian response			Normal ovarian response			High ovarian response		
	CLBR positive	CLBR negative	P-Value	CLBR positive	CLBR negative	P-Value	CLBR positive	CLBR negative	*P*-Value
E_2_(Gn1), pg/mL	40(22,52)	48(37,63)	0.081	42(30,58)	42(28.5,57)	0.763	38.54(31,49)	44(26.15,53)	0.024
E_2_(Gn5), pg/mL	260(214,297)	305(210,440)	0.131	572(324,917)	519(350,1033.5)	0.843	681(406,1468)	913(562,1637)	0.014*
E_2_(Gn8), pg/mL	708(589,1111)	797(541,1137)	0.702	1780(1100,2917)	1697(1187,2490)	0.781	3338(1889,4800)	3854(2115,4800)	0.343
E_2_(HCG), pg/mL	1263(928,1898)	1283(970,1650)	0.924	3887(2219,4982)	3867(2619,4870)	0.956	4870(4525,5010)	5010(4800,5010)	< 0.001*
E_2_ ratio of Gn5/Gn1	7.82(3.72,12.33)	6.07(4.22,9.18)	0.45	12.64(8.02,24.33)	11.30(7.37,23.19)	0.441	19.8(10.5,38.12)	23.35(9.35,38.1)	0.491
E_2_ ratio of Gn8/Gn5	3.06(2.92,3.58)	2.6(2.08,2.95)	< 0.001*	3.33(2.65,3.84)	3.11(2.55,3.83)	0.017*	3.43(2.77,5.02)	3.54(2.32,4.38)	0.004*

Limits of high and low ovarian response used in the current analyses were set at > 18 oocytes retrieved and < 6 oocytes retrieved, respectively

### Effects of estradiol ratios on IVF-ET outcome

As indicated in Table 7, logistic regression analysis revealed that both group A1 (OR = 0.376 [0.182–0.779]; *P* = 0.008*, OR = 0.401 [0.188–0.857]; *P* = 0.018*) and B1 (OR = 0.363 [0.179–0.735]; *P* = 0.005*, OR = 0.389 [0.187–0.808]; *P* = 0.011*) had opposite influences on CCPR and CLBR compared with groups A4 and B4, whereas groups A3 were weakly but significantly associated with CCPR.

**Table 7:** Binary logistic regression on CCPR and CLBR with the ratio of serum E_2_

			95% CI					95% CI		
Dependent variable: CCPR	*P*-Value	OR	Lower	Upper	Dependent variable: CLBR	*P*-Value	OR	Lower	Upper	
Independent variables					Independent variables					
A	0.043*				A	0.099				
A1	0.008*	0.376	0.182	0.779	A1	0.018*	0.401	0.188	0.857	
A2	0.299	0.699	0.356	1.374	A2	0.557	0.816	0.414	1.609	
A3	0.040*	0.495	0.253	0.969	A3	0.207	0.647	0.329	1.272	
B	0.013*				B	0.015*				
B1	0.005*	0.363	0.179	0.735	B1	0.011*	0.389	0.187	0.808	
B2	0.086	0.559	0.287	1.086	B2	0.067	0.526	0.265	1.047	
B3	0.911	0.964	0.504	1.842	B3	0.860	1.060	0.556	2.021	

The independent variables also included female age, BMI, duration of infertility, and number of high-quality embryos.. We defined the group A4 and B4 as the last reference category

Abbreviations: *OR * Odds ratio, *CI * Confidence interval

## Discussion

The role of E_2_ in IVF-ET is well known to the stage of the trigger day of hCG injection, and it indicates follicular maturation when estrogen level reaches 250 pg/mL; however, its role before that stage remains controversial. In this study, the serum estrogen levels of Gn1, Gn5, Gn8, and HCG were measured, and their ratio was calculated to evaluate ovarian response and predict treatment outcomes. In the statistical analysis, the estrogen levels of Gn5, Gn8, and HCG, as well as the ratios of Gn5/Gn1, Gn8/Gn5, and HCG/Gn1, all had clinical guiding significance, and the low growth level and ratio significantly reduced CCPR and CLBR. The increment coefficient of estrogen was observed at different stages of IVF-ET, and it was discovered that during Gn stimulation, the change in estrogen in the early and middle stages was also associated with pregnancy outcome, which may be linked to the follicle growth mode. During the early follicular stage, a group of antral follicles is recruited and induced to develop. The collected follicular fluid had low estrogen levels. At this point, estrogen growth may be linked to recruited follicles. As the follicle grows, follicular granulosa cells increase in number and exhibit aromatase activity [35]. Therefore, follicular fluid contains high levels of estrogen. At this stage, the increase in estrogen levels may be related to follicular quality.

Second, no study has explored the relationship between the ratio of E_2_ increase during Gn treatment and prognosis with an antagonist regimen. According to Table 2, we revealed that serum E_2_ ratios of groups A (Gn5/Gn1) and B (Gn8/Gn5) were statistically significant compared with pregnancy outcomes. Therefore, we chose these two indicators for further analysis and grouping according to the 25th, 50th, and 75th percentiles. The Chi-square test and constructing a binary logistic regression analysis model aimed at pregnancy outcomes revealed that patients with lower serum E_2_ ratios in groups A1 and B1 had lower CCPR and CLBR, and group B had more significance. It is suggested that, in clinical medication, estrogen levels can be observed after five days of Gn treatment, and medication can be adjusted when the increase in estrogen is not ideal to obtain satisfactory efficacy.

Third, we analyzed the factors influencing the E_2_ ratio during the Gn stimulation cycle. According to the statistical analysis, the increase in estrogen levels during the middle stage of Gn stimulation (Gn8/Gn5, group B) was associated with age, infertility years, and BMI but not with the increase in estrogen levels during the early stage of Gn stimulation (Gn5/Gn1, group A). These results imply that the basic characteristics of patients greatly affect the rate of estrogen increase during the Gn8/Gn5 stage and may be a key factor affecting follicle quality. In follicular growth, a higher ratio of estrogen increase can result in higher clinical pregnancies and live births, regardless of whether they are in groups A or B. However, the link between groups A and B was the opposite. The estrogen ratio in group B decreased as the ratio in group A increased. Estrogen growth was fastest in the early stage and slowest in the middle stage in the corresponding patients. An insufficient increase in estrogen levels in patients who recruit more follicles in the early stage could be due to an insufficient dose of Gn. When the association between group A and Gn usage was examined, it was discovered that the longer the Gn days and the higher the dose of Gn, the slower the early-stage estrogen increase. This may be related to the patient’s baseline conditions. Typically, patients with a high BMI, age, or more antral follicles receive a higher Gn initiation dosage, although a lower estrogen growth rate is generally obtained. In contrast, in the group B study, the longer the Gn days and the larger the required dosage of Gn, the faster estrogen growth in the middle stage. This may indicate the regularity of Gn dosage in follicular development process, and the dosage of Gn8/Gn5 is more critical at this stage, and it is also the time to increase Gn dosage in a clinical setting.

Fourth, the main purpose of monitoring estrogen in IVF-ET is to assess the availability of adequate quantity and quality of mature oocytes on the trigger day. This study revealed that estrogen ratios increased during early and middle ovulation induction (Gn5/Gn1, Gn8/Gn5), as well as estrogen levels on hCG day (HCG), were significant for IVF-ET outcome. However, the ratio of estrogen increase was not significantly different in the late stages of Gn-stimulated follicular growth (HCG/Gn5 and HCG/Gn8). Tan et al. [36] discovered no statistically significant differences in pregnancy rates among three groups of patients who respectively received hCG on the day of the leading follicle reaching 18 mm, on the second day, and the third day. At the moment of follicular maturation, estrogen concentration may be more important than estrogen growth ratio, necessitating a rethink of the role of estrogen in trigger day selection.

Predicting outcomes in ART may allow for earlier treatment strategy adjustment and protect patients from unnecessary physical and financial burdens throughout the treatment cycle. It is currently being monitored using recurrent transvaginal ultrasonography or serum E_2_. We considered that ultrasound could measure follicle growth, whereas serum E_2_ levels mainly reflected follicle function. As a result, estradiol plays a critical role despite its relatively low predictive value as a single factor. Additional parameters are required to identify more sensitive biochemical markers that may predict the probability of achieving clinical pregnancy before hCG administration. Our study demonstrated that accurate monitoring of E_2_ ratios is a key aspect that supports the prognosis of IVF-ET outcomes, dose adjustment, and cycle cancellation evaluation. This provides the clinic with a new, simple, and convenient predictive method. By observing and calculating the range of E_2_ increase ratio, we found that both Gn5/Gn1 and Gn8/Gn5 could predict IVF outcome success, but Gn8/Gn5 was more accurate. Maintaining a serum estradiol increase ratio above 6.44 on Gn5/Gn1 and 2.39 on Gn8/Gn5 may result in a higher pregnancy rate.

The main limitation of our study was related to the sample size. It was difficult to recover all the necessary data for calculating the estradiol increase ratios throughout the stimulation treatment. Therefore, we excluded patients with insufficient information and those who had achieved pregnancy but had not yet given birth. Furthermore, we need to evaluate whether using different stimulation gonadotropin doses will lead to a deviation in our results in a single center.

## Data Availability

The datasets used and analyzed during the current study are available from the corresponding author upon reasonable request**.**

## Abbreviations

hCG: Human chronic gonadotropin
IVF-ET: In vitro fertilization-embryo transfer
Gn: Gonadotropin
ART: Assisted reproductive technology
E_2_: Estradiol
BMI: Body mass index
Gn days: Gonadotropin days
Gn dosage: Gonadotropin dosage
Gn1: Serum estradiol on the day of gonadotrophin
Gn5: Serum estradiol on the five days after gonadotropin stimulation
Gn8: Serum estradiol on the eight days after gonadotropin stimulation
HCG: Serum estradiol on the trigger day of human chorionic gonadotropin injection
Gn5/Gn1: The serum estradiol levels of Gn5 divided by Gn1
Gn8/Gn5: The serum estradiol levels of Gn8 divided by Gn5
HCG/Gn1: The serum estradiol levels of HCG divided by Gn1
HCG/Gn5: The serum estradiol levels of HCG divided by Gn5
HCG/Gn8: The serum estradiol levels of HCG divided by Gn8
CCPR: Cumulative clinical pregnancy rate
CLBR: Cumulative live birth rate
